# Inflammation‐induced left ventricular fibrosis is partially mediated by tumor necrosis factor‐α

**DOI:** 10.14814/phy2.15062

**Published:** 2021-10-29

**Authors:** Ashmeetha Manilall, Lebogang Mokotedi, Sulè Gunter, Regina Le Roux, Serena Fourie, Colleen A. Flanagan, Aletta M.E. Millen

**Affiliations:** ^1^ Cardiovascular Pathophysiology and Genomics Research Unit School of Physiology Faculty of Health Sciences University of the Witwatersrand Johannesburg South Africa; ^2^ Molecular Physiology Laboratory School of Physiology Faculty of Health Sciences University of the Witwatersrand Johannesburg South Africa

**Keywords:** diastolic dysfunction, gene expression, inflammation, myocardial fibrosis, tumor necrosis factor alpha inhibitor

## Abstract

**Objective:**

To determine the mechanisms of inflammation‐induced left ventricular (LV) remodeling and effects of blocking circulating tumor necrosis factor alpha (TNF‐α) in a model of systemic inflammation.

**Methods:**

Seventy Sprague‐Dawley rats were divided into three groups: the control group, the collagen‐induced arthritis (CIA) group, and the anti‐TNF‐α group. Inflammation was induced in the CIA and anti‐TNF‐α groups. Following the onset of arthritis, the anti‐TNF‐α group received the TNF‐α inhibitor, etanercept, for 6 weeks. LV geometry and function were assessed with echocardiography. Circulating inflammatory markers were measured by ELISA and LV gene expression was assessed by comparative TaqMan® polymerase chain reaction.

**Results:**

The LV relative gene expression of pro‐fibrotic genes, transforming growth factor β (*TGFβ*) (*p *= 0.03), collagen I (*Col1*) (*p *< 0.0001), and lysyl oxidase (*LOX*) (*p *= 0.002), was increased in the CIA group compared with controls, consistent with increased relative wall thickness (*p *= 0.0009). *Col1 and LOX* expression in the anti‐TNF‐α group were similar to controls (both, *p* > 0.05) and tended to be lower compared to the CIA group (*p* = 0.06 and *p* = 0.08, respectively), and may, in part, contribute to the decreased relative wall thickness in the anti‐TNF‐α group compared to the CIA group (*p* = 0.03). In the CIA group, the relative gene expression of matrix metalloproteinase 2 (*MMP2*) and *MMP9* was increased compared to control (*p* = 0.04) and anti‐TNF‐α (*p* < 0.0001) groups, respectively.

**Conclusion:**

Chronic systemic inflammation induces fibrosis and dysregulated LV extracellular matrix remodeling by increasing local cardiac pro‐fibrotic gene expression, which is partially mediated by TNF‐α. Inflammation‐induced LV diastolic dysfunction is likely independent of myocardial fibrosis.

## Graphical abstract

High‐grade systemic inflammation increases macrophage infiltration, pro‐fibrotic gene expression, and upregulates extracellular matrix turnover within left ventricular tissue. Inhibition of circulating TNF‐α, with the anti‐inflammatory drug etanercept, partially attenuates pro‐fibrotic gene expression and reduces extracellular matrix turnover, thereby preventing myocardial fibrosis and adverse left ventricular remodeling. Inflammation‐induced left ventricular diastolic dysfunction is likely independent of myocardial fibrosis.

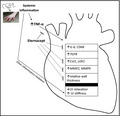

## 1 INTRODUCTION

Left ventricular (LV) diastolic dysfunction is a pre‐clinical condition that often progresses to heart failure with a preserved ejection fraction (HFpEF) (Wan et al., 2014). Although low‐grade systemic inflammation is believed to be a causal mechanism underlying LV diastolic dysfunction development in patients with comorbid conditions such as diabetes mellitus, obesity, and hypertension, other factors in these conditions confound understanding of the contribution of inflammation to the development of LV diastolic dysfunction (Van Linthout & Tschope, 2017). Patients with autoimmune conditions characterized by chronic systemic inflammation have increased risk for developing LV diastolic dysfunction (Aslam et al., 2013). Indeed, models of systemic inflammation provide useful information about the mechanisms whereby inflammation contributes to the development of diastolic dysfunction (Glezeva & Baugh, 2014).

Myocardial fibrosis contributes to concentric remodeling of the LV, which is characteristic of diastolic dysfunction. Myocardial fibrosis is characterized by extracellular matrix changes and hypertrophic growth of cardiomyocytes (Baum & Duffy, 2011). Several factors including increased collagen volume, collagen cross‐linking, and disruption of collagen turnover contribute to the formation of a stiffened extracellular matrix (El Hajj et al., 2017). Inflammation modulates the mechanisms of extracellular matrix remodeling and may thus contribute to the development of diastolic dysfunction (Goldsmith et al., 2013; Gu et al., 2015). Increased expression of pro‐inflammatory cytokines has been associated with accelerated collagen formation (Porter & Turner, 2009), increased collagen cross‐linking (Suthahar et al., 2017), and altered collagen degradation, which ultimately lead to dysregulation of collagen turnover and LV fibrosis (Li et al., 2000). Although low‐grade inflammation induced by comorbidities has been associated with these mechanisms of myocardial fibrosis and remodeling (Paulus & Tschope, 2013), the effect of inflammation on these mechanisms of the LV remodeling and diastolic dysfunction, in the absence of confounding comorbidities is uncertain.

Anti‐inflammatory drug treatment in auto‐immune conditions characterized by systemic inflammation is known to reduce disease progression. The effects of these drugs on myocardial remodeling and diastolic dysfunction have been contradictory (Kosmas et al., 2019). Some have shown that blocking circulating inflammatory cytokines positively impacts the cellular mechanisms implicated in myocardial remodeling (Kleveland et al., 2016; Li et al., 2017), while others have failed to show positive outcomes (Hartman et al., 2016). Nevertheless, the beneficial effects were shown in systems that did not involve chronic inflammation (Kleveland et al., 2016; Li et al., 2017). We have recently shown that systemic inflammation‐induced concentric remodeling, diastolic dysfunction (Le Roux et al., 2021; Mokotedi et al., 2020), and increased collagen area fraction (Mokotedi et al., 2020) in the collagen‐induced arthritis (CIA) rat model. The aim of this study was to assess the molecular mechanisms of inflammation‐induced cardiac fibrosis in diastolic dysfunction and to use the tumor necrosis factor‐α (TNF‐α) inhibitor, etanercept, to assess the role of circulating TNF‐α in the development of fibrosis.

## 2 MATERIALS AND METHODS

### 2.1 Animal treatment and sample collection

Experimental procedures were approved by the Animal Ethics Screening Committee of the University of the Witwatersrand (AESC numbers: 2017/03/21C and 2019/02/10C) and conducted in compliance with the Guide for the Care and Use of Laboratory Animals. Three‐month‐old Sprague‐Dawley rats were randomly assigned to three groups. Rats in the control group (*n* = 27, males *n* = 15; females *n* = 12) received a subcutaneous injection of saline (0.2 ml), at the base of the tail. To induce systemic inflammation, the CIA group (*n* = 28, males *n* = 13; females *n* = 15) and the TNF‐α inhibitor group (anti‐TNF‐α, *n* = 15, males *n* = 7; females *n* = 8) were injected subcutaneously, at the base of the tail, with bovine type II collagen (2 mg/ml in 0.05 M acetic acid) emulsified in incomplete Freund's adjuvant (1:1 volume, 0.2 ml), as previously described (Le Roux et al., 2021). A booster injection (0.1 ml) was given at 7 and 21 days after the first immunization. All collagen‐treated rats exhibited paw inflammation as previously described (Le Roux et al., 2021). After development of inflammation, the anti‐TNF‐α group received etanercept (10 mg/kg, every 3 days) for 6 weeks. Blood pressure was measured as previously described (Le Roux et al., 2021). For this study, 70 rats that are part of a larger study in which echocardiography was performed (Le Roux et al., 2021), were used for assessment of LV gene expression of markers of fibrosis and extracellular matrix turnover.

### 2.2 Echocardiography

After 6 weeks of treatment, rats were anesthetized by intramuscular injections of ketamine (100 mg kg^−1^) and xylazine (5 mg kg^−1^) to perform echocardiography using an ultrasound (Acuson SC 2000, Siemens Medical Solutions, USA, Inc.), as previously described (Le Roux et al., 2021). Briefly, LV geometry was determined by two‐dimensional M‐mode images according to convention. Concentric LV remodeling was determined by calculating relative wall thickness. Mitral annular tissue lengthening was measured with tissue Doppler imaging, in the apical four chamber view during early (e') and late (a') diastole. LV stiffness was expressed as the lateral wall e'/a' ratio.

Subsequently, rats were terminated by thoracotomy and hearts were removed. The heart was dissected and stored (−80°C) in RNA*later®* (Ambion, Sigma Aldrich). Whole blood was collected, and serum was aliquoted and frozen (−80°C).

### 2.3 Circulating inflammatory markers

TNF‐α, interleukin (IL)‐6, and C‐reactive protein (CRP) concentrations in serum were measured in duplicate by sandwich enzyme‐linked immunosorbent assay (ELISA, Elabscience Biotechnology) (Le Roux et al., 2021).

### 2.4 Preparation of cDNA and comparative gene expression using real‐time PCR

Rat LV samples were homogenized by sonication and total RNA was extracted using an illustra™ Mini Spin RNA extraction kit (GE Healthcare, Buckinghamshire). The resulting RNA was reverse transcribed to produce cDNA using SuperScript™ IV VILO™ cDNA Synthesis Master Mix (Thermo Fisher Scientific, Life Technologies).

Comparative gene expression RT‐PCR was performed using a StepOne Plus thermocycler (Thermo Fisher Scientific, Life Technologies) with cDNA (~2 µg), pre‐designed TaqMan probe mixes for the reference gene *HPRT1* (0.25 µl, Rn01527838_g1, Thermo Fisher Scientific, Life Technologies), pre‐designed TaqMan probe mixes for each gene of interest (0.5 µl, TaqMan gene expression assay), and TaqMan Advanced PCR Master Mix (5 µl) in a final volume of 10 µl. Comparative gene expression of the following genes was assessed; *Col1* (Rn01463848_m1), *TGFβ* (Rn99999016_m1), *LOX* (Rn01491829_m1), *TNF*‐*α* (Rn00562055_m1), *IL*‐*6* (Rn01410330_m1), α‐smooth muscle actin (*α*‐*SMA*, Rn01759928_g1), *CD68* (Rn01495634_g1), myosin heavy chain β (*Myh7*, Rn00568328_m1), *MMP2* (Rn01538170_m1), and *MMP9* (Rn00579162_m1) in duplex reactions with *HPRT1* (0.25µl) in duplicated plates. The fold change in relative expression was then calculated as 2^−∆∆Ct^, using the delta‐delta Ct method (Livak & Schmittgen, 2001).

### 2.5 Data analysis

Statistical analyses were performed using SAS software, version 9.4 (SAS Institute Inc.,). Normally distributed variables are expressed as mean ± SEM and non‐normally distributed variables are expressed as median (interquartile range, IQR). Differences in normally distributed variables between the groups were determined by two‐way analysis of variance (ANOVA) with group and sex as the main effects, followed by Tukey's post hoc tests. For non‐normally distributed variables, group differences were determined by Kruskal–Wallis tests. As there were no differences in the molecular markers between males and females (Table S1), results were combined. Associations were determined using Pearson's correlations. To determine the independent contribution of molecular markers to LV remodeling, linear regression analysis was performed. Non‐normally distributed variables were log transformed prior to inclusion in regression analysis. Results were considered statistically significant if *p* ≤ 0.05.

## 3 RESULTS

### 3.1 Effect of collagen inoculation and anti‐TNF‐α treatment on body mass, blood pressure, and circulating and tissue inflammatory markers

The characterization of the animal model has been reported (Le Roux et al., 2021). Briefly, for the animals included in this study, body mass was lower in the anti‐TNF‐α (*p* = 0.001) group, but not in the CIA group (*p* = 0.07) compared to controls (Table 1). Blood pressure was similar between groups (Table 1). Circulating inflammatory marker concentrations were higher in both groups of rats inoculated with collagen compared to the control group (Table 1). Circulating CRP concentrations were lower in the anti‐TNF‐α group compared to the CIA group (*p* = 0.02).

**TABLE 1:** Body mass, blood pressure, and circulating and tissue inflammatory markers in control, CIA, and anti‐TNF‐α groups

	Control	CIA	anti‐TNF‐α
*n*	27	28	15
Body mass (g)	455.7 ± 9.5	425.8 ± 9.1	404.8 ± 12.4*
SBP (mm Hg)	128 ± 2	130 ± 2	131 ± 2
DBP (mm Hg)	88 ± 2	88 ± 1	85 ± 1
Circulating inflammatory marker concentrations			
TNF‐α (pg/ml)	91.3 ± 6.7	159.4 ± 6.4*	143.3 ± 8.4*
IL‐6 (pg/ml)	15.9 ± 1.9	30.8 ± 1.9*	26.8 ± 2.5*
CRP (ng/ml)	0.09 ± 0.04	0.58 ± 0.04*	0.41 ± 0.05* ^,^ **
Tissue inflammatory marker expressions (mRNA)			
*TNF‐α*	1.02 (0.71–1.27)	1.03 (0.85–1.28)	1.14 (1.04–1.47)
*IL‐6*	0.85 (0.65–0.93)	1.54 (0.86–2.22)*	1.04 (0.86–1.28)
*CD68*	0.92 ± 0.05	1.12 ± 0.06*	0.96 ± 0.07

Data are expressed as means ± SEM or median (IQR).

Abbreviations: anti‐TNF‐α, collagen‐inoculated and treated with tumor necrosis factor alpha inhibitor; CIA, collagen‐induced arthritis; CRP, C‐reactive protein; DBP, diastolic blood pressure; IL‐6, interleukin 6; SBP, systolic blood pressure; TNF‐α, tumor necrosis factor alpha.

* *p* < 0.05 versus control

** *p* < 0.05 versus CIA.

Collagen inoculation did not affect LV relative mRNA expression of *TNF‐α* (Table 1). The LV relative mRNA expression of *IL‐6* was higher in the CIA group (*p* = 0.001) than in controls (Table 1). Relative mRNA expression of *IL‐6* was similar in the anti‐TNF‐α and control groups (*p* = 0.06). Compared to the control group, the relative mRNA expression of *CD68*, a marker of monocytes and macrophages present in the LV tissue, was higher in the CIA group (*p* = 0.03), but not in the anti‐TNF‐α group (*p* = 0.92).

### 3.2 Effect of collagen inoculation and anti‐TNF‐α treatment on extracellular matrix gene expression and LV remodeling

**FIGURE 1:**
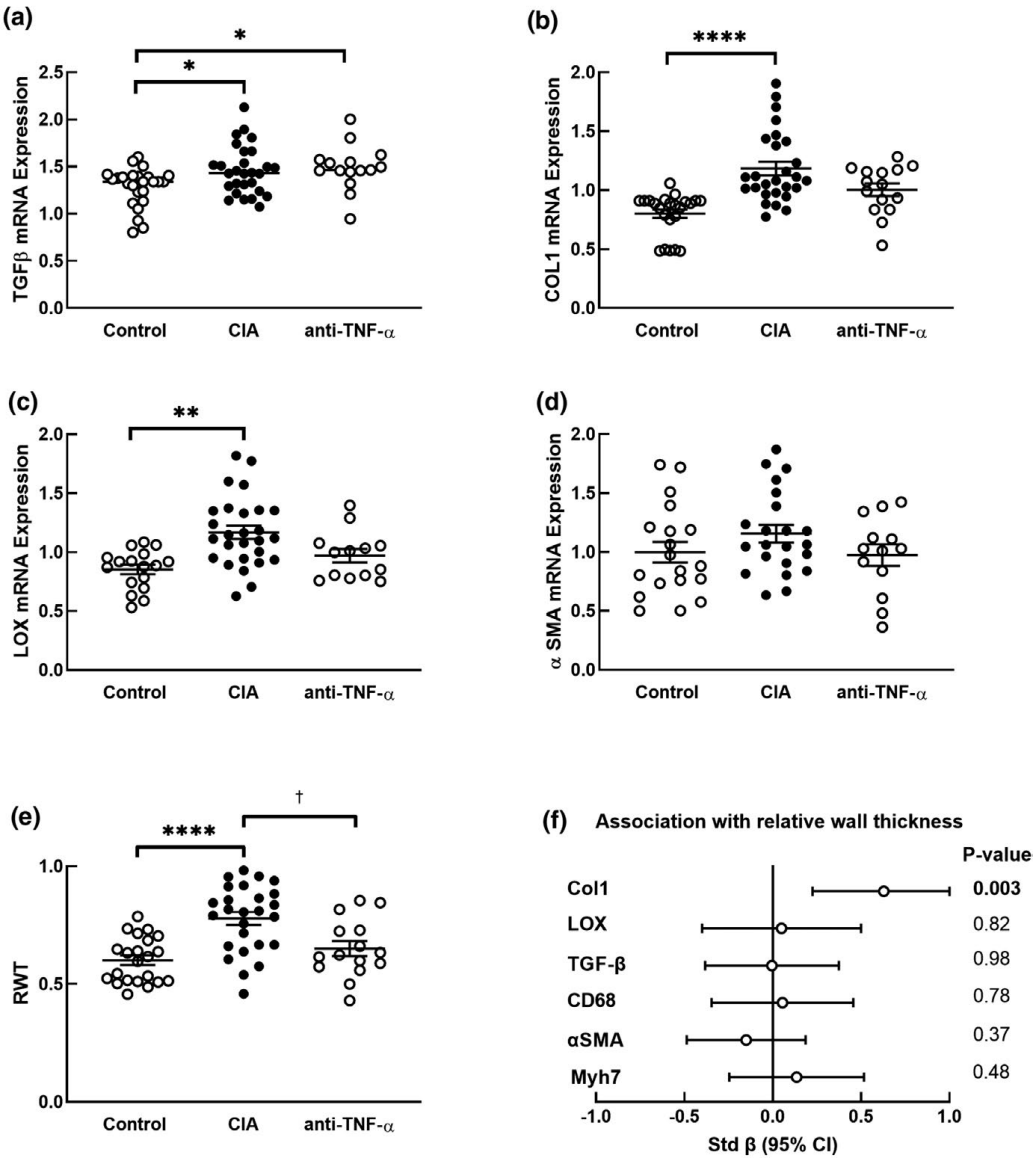
LV geometry and extracellular matrix gene expression. Relative mRNA expression of *TGFβ* (a), *Col1* (b), *LOX* (c), *α*‐*SMA* (d), echocardiographic measurement of relative wall thickness (RWT) of the left ventricles (e). Group differences are determined by two‐way analysis of variance (ANOVA) with group and sex as the main effects, followed by Tukey's post hoc tests. In (a)–(d), results are expressed in arbitrary units normalized for the expression of the endogenous reference gene, *HPRT*. Data are presented as means ± SEM (a‐e); * *p* < 0.05 versus control; ** *p* < 0.01 versus control; ***p < 0.001 versus control; ****p < 0.0001 versus control; ^†^ *p* < 0.05 versus CIA. Contribution of *Col1*, *LOX*, *TGFβ*, *CD68*, *α*‐*SMA*, and *Myh7* in left ventricular tissues to relative wall thickness is (f) determined by linear regression. Open circles represent standardized beta coefficients and error bars represent 95% CI

The relative mRNA expression of *TGFβ*, which codes for the protein that stimulates fibrosis in LV tissue, was higher in CIA (*p* = 0.03) and anti‐TNF‐α (*p* = 0.03) groups compared to the control group (Figure 1a). Treatment with etanercept did not impact the increase in relative mRNA expression of *TGFβ* that was induced by collagen inoculation (*p* = 0.92; Figure 1a). Since *TGFβ* is primarily produced by monocytes in the resolution phase of inflammation (Netea et al., 2017), we assessed the association between the expression of *TGFβ* and expression of the monocyte marker, *CD68*. The relative mRNA expression of *TGFβ* was not associated with *CD68* expression (r [95% CI] = −0.004 [−0.27 to 0.28], *p* = 0.98).

**FIGURE 2:**
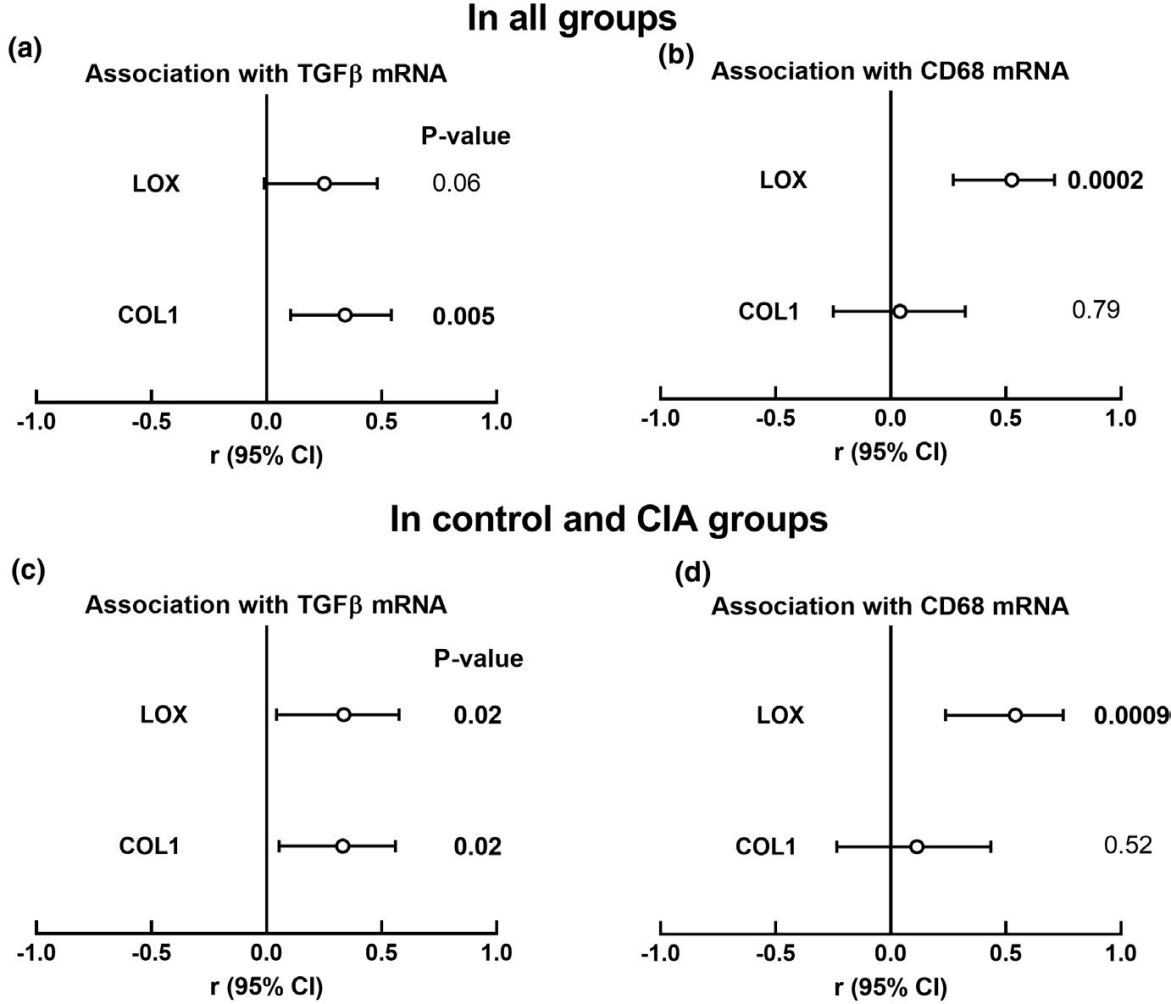
*LOX* and *COL1* markers of ECM gene expression. Pearson's correlations of *TGFβ* and *CD68* with *Col1* and *LOX* in all groups (a, b) and control versus CIA groups only (c, d). Open circles represent correlation coefficients and error bars represent 95% CI

The relative mRNA expression of *Col1*, which codes for the extracellular matrix protein collagen 1, was higher in the CIA group (*p* < 0.0001), but not in the anti‐TNF‐α (*p* = 0.07) group compared to the control group (Figure 1b). The expression of *Col1* was lower in the anti‐TNF‐α group than in the CIA group but did not reach statistical significance (*p* = 0.06). Consistent with the function of TGFβ in stimulating expression of collagen (Mollmann et al., 2010), LV *Col1* expression was correlated with LV *TGFβ* expression in all groups (*p* = 0.005; Figure 2a) and in the control and CIA groups (*p* = 0.02; Figure 2c). Since monocytes are the major source of *TGFβ*, we assessed the association between *Col1* expression and *CD68* expression. LV *Col1* expression was not associated with *CD68* expression in all groups (*p* = 0.79, Figure 2b) or in the control and inflammation groups (*p* = 0.52; Figure 2d), consistent with no association of *TGFβ* expression with *CD68* expression.

Compared to the control group, relative mRNA expression of *LOX*, which codes for the enzyme that catalyzes collagen cross‐linking, was higher in the CIA group (*p* = 0.002), but not in the anti‐TNF‐α group (*p* = 0.26, Figure 1c). The expression of *LOX* was lower in the anti‐TNF‐α group than in the CIA group but did not reach statistical significance (*p* = 0.08). This suggests that blocking circulating TNF‐α reduces inflammation‐induced upregulation of *LOX* expression. Since *LOX* is expressed in fibroblasts that are regulated by *TGFβ*, which is produced by monocytes, we assessed the association between *LOX* expression and the expression of *CD68* and *TGFβ*. The relative mRNA expression of *LOX* was associated with *CD68*, in all groups (*p* = 0.0002; Figure 2b) and in the control and CIA groups only (*p* = 0.0009; Figure 2d). The relative mRNA expression of *LOX* was associated with the relative mRNA expression of *TGFβ* in the control and CIA groups (*p* = 0.02; Figure 2c), but not when all groups were included in the analysis (*p* = 0.06) (Figure 2a). There were no differences between the groups in the LV relative mRNA expression of *α*‐*SMA* (Figure 1d).

The LV relative wall thickness (RWT) was higher in the CIA group than in the control group (*p* = 0.0009, Figure 1e), showing that collagen inoculation‐induced concentric remodeling. The LV RWT in the anti‐TNF‐α‐treated group was lower than in the CIA group (*p* = 0.03) and was not different from controls (*p* = 0.77, Figure 1e). The mean ± SEM LV relative expression of *Myh7* was higher in the CIA group (1.25 ± 0.05) compared to the control group (0.97 ± 0.05; *p* = 0.0005), showing collagen inoculation‐induced LV hypertrophy. The expression of *Myh7* was lower in the anti‐TNF‐α group (0.91 ± 0.06) compared to the CIA group (*p* = 0.0002) and was not different from controls (*p* = 0.74). When determining the independent contribution of pro‐fibrotic and hypertrophy gene expression markers to RWT, only *Col1* was associated with an increased RWT (std β (95% CI) = 0.62 (0.22–1.02), *p* = 0.003; Figure 1F).

### 3.3 Effect of collagen inoculation and anti‐TNF‐α treatment on markers of collagen turnover and LV relaxation

**FIGURE 3:**
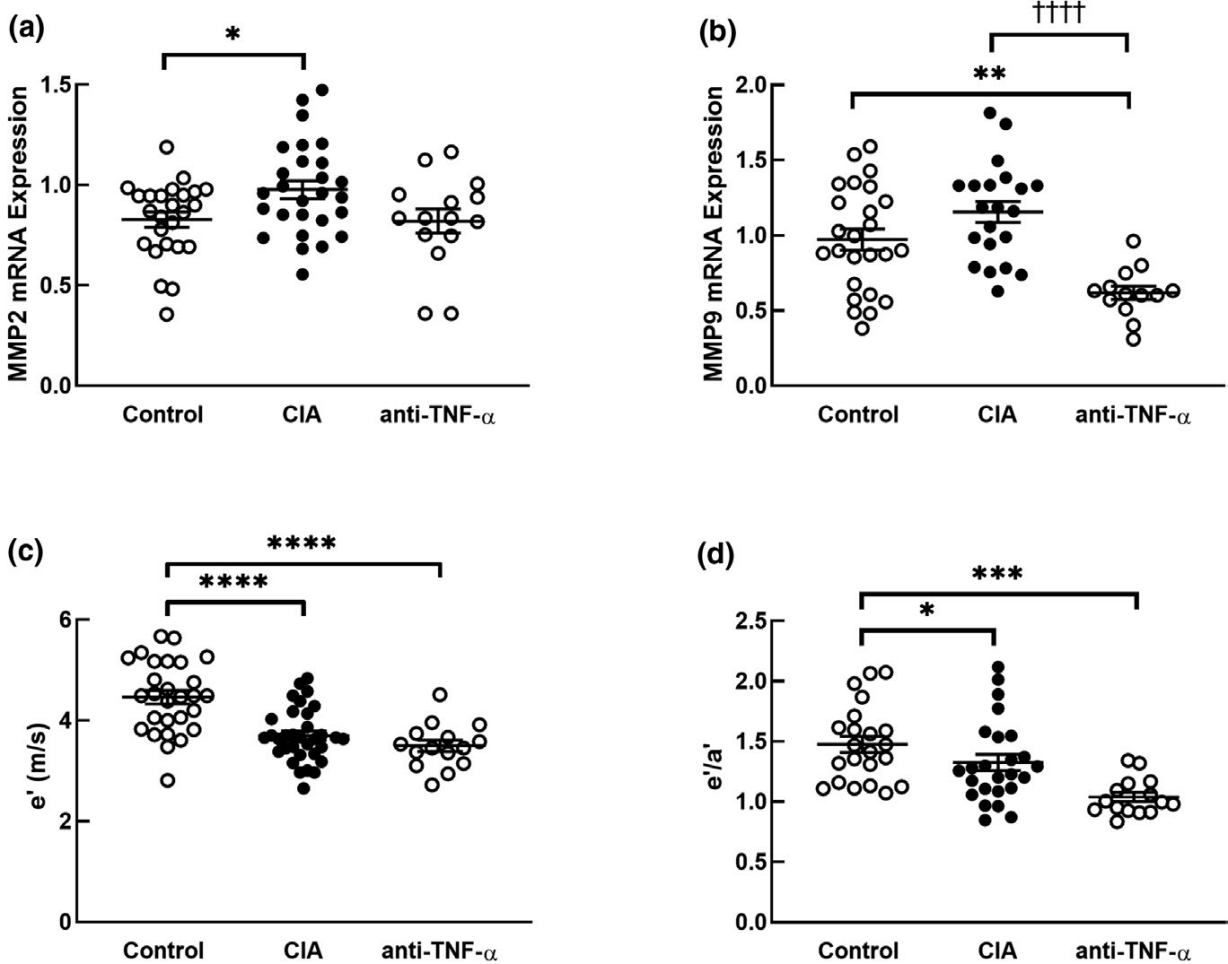
Expression of matrix metalloproteinases and measures of diastolic dysfunction. Relative mRNA expression of *MMP2* (a) and *MMP9* (b) in left ventricular tissues and echocardiographic measures, e’ (c) and e’/a’ (d) of control, CIA, and anti‐TNF‐α rats. Group differences are determined by two‐way analysis of variance (ANOVA) with group and sex as the main effects, followed by Tukey's post hoc tests. mRNA expression data expressed in arbitrary units normalized for the expression of the endogenous reference gene, *HPRT1*. Data are presented as means ± SEM; *p < 0.05 versus control; **p < 0.01 versus control; ***p < 0.001 versus control; ****p < 0.0001 versus control; ^††††^ *p* < 0.0001 versus anti‐TNF‐α

The LV relative mRNA expression of *MMP2*, which codes for a protein that degrades ECM components and contributes to collagen turnover, was higher in the CIA group than in the control group (*p* = 0.04, Figure 3a). The LV relative mRNA expression of *MMP2* in the anti‐TNF‐α group tended to be lower than the CIA group (*p* = 0.07) and was similar to the control group (*p* = 0.99). Since matrix metalloproteinases play an important role in regulating collagen turnover, and inflammation resulted in increased collagen production, it is not clear whether matrix metalloproteinase expression is likely to be stimulated by systemic inflammation or by local fibrosis. Therefore, we assessed the association between *MMP2* expression and circulating inflammatory proteins and local LV expression of cytokines and pro‐fibrotic genes. The LV relative mRNA expression of *MMP2* was not associated with circulating IL‐6 (r [95% CI], 0.10 [−0.16 to 0.35]; *p* = 0.45), TNF‐α (r [95% CI], 0.10 [−0.16 to 0.34]; *p* = 0.46), or CRP (r [95% CI], 0.07 [−0.19 to 0.32]; *p* = 0.61) concentrations (Table 2). The *MMP2* expression was associated with local LV expression of *IL*‐*6* (r [95% CI], 0.42 [0.15–0.62]; *p* = 0.002), *TGFβ* (r [95% CI], 0.48 [0.27–0.64]; *p* < 0.0001), and *Col1* (r [95% CI], 0.49 [0.28–0.66]; *p* < 0.0001) (Table 2).

**TABLE 2:** Association between expression of matrix metalloproteinases and pro‐inflammatory and pro‐fibrotic genes

	*MMP2* versus		*MMP9* versus	
	r (95% CI)	*p*	r (95% CI)	*p*
Circulating inflammatory proteins				
CRP	0.07 (−0.19 to 0.32)	0.61	0.22 (−0.07 to 0.47)	0.13
IL‐6	0.10 (−0.16 to 0.35)	0.45	0.13 (−0.15 to 0.40)	0.35
TNF‐α	0.10 (−0.16 to 0.34)	0.46	0.09 (−0.20 to 0.36)	0.55
Tissue inflammation genes				
*IL‐6*	**0.42 (0.15–0.62)**	**0.002**	0.21 (−0.09 to 0.48)	0.16
*TNF‐α*	−0.15 (−0.39 to 0.12)	0.28	0.08 (−0.20 to 0.36)	0.57
Tissue fibrosis genes				
*LOX*	0.21 (−0.06 to 0.44)	0.13	**0.34 (0.06 to 0.56)**	**0.02**
*Col1*	**0.49 (0.28 to 0.66)**	**<0.0001**	−0.15 (−0.41 to 0.12)	0.27
Genes expressed by inflammatory cells				
*TGFβ*	**0.48 (0.27 to 0.64)**	**<0.0001**	−0.03 (−0.28 to 0.24)	0.84
*CD68*	0.02 (−0.27 to 0.30)	0.91	0.20 (−0.11 to 0.47)	0.19

Data are expressed as Pearson's r (95% CI).

Abbreviations: CI, confidence interval; Col1, collagen I; CRP, C‐reactive protein, IL‐6, interleukin 6; LOX, lysyl oxidase; MMP2, matrix metalloproteinase 2; MMP9, matrix metalloproteinase 9; TGF‐β, transforming growth factor beta. TNF‐α, tumor necrosis factor‐α.

Significant values are shown in bold.

The LV relative mRNA expression of *MMP9*, was significantly lower in the anti‐TNF‐α group compared to the control (*p* = 0.001) and CIA (*p* < 0.0001) groups (Figure 3b), showing that blocking circulating TNF‐α decreases LV expression of *MMP9*. We also assessed the correlation of *MMP9* expression with circulating inflammatory proteins and local LV expression of cytokines and pro‐fibrotic genes. The local LV relative mRNA expression of *MMP9* was only associated with *LOX* (r [95% CI], 0.34 [0.06–0.56]; *p* = 0.02) (Table 2).

Since matrix metalloproteinases increase extracellular matrix turnover, altered expression would be expected to affect myocardial stiffness. LV relaxation, as indexed by a decreased e', was impaired in both the CIA (*p* < 0.0001) and anti‐TNF‐α (*p* < 0.0001) groups compared to the control group (Figure 3c). LV lateral wall e'/a' ratio was decreased in the CIA group (*p* = 0.04) and the anti‐TNF‐α group (*p* = 0.0001) compared to the control group (Figure 3d), indicating increased LV stiffness in both groups inoculated with collagen.

## 4 DISCUSSION

This study used a CIA model of systemic inflammation to investigate the molecular mechanisms of inflammation‐induced diastolic dysfunction, focusing on LV fibrosis and extracellular matrix remodeling. We showed that systemic inflammation resulted in local LV tissue inflammation, as evidenced by increased LV expression of *IL‐6* and inflammatory leukocyte infiltration, which were inhibited when circulating TNF‐α was blocked. The LV expression of *TGFβ* was increased in animals inoculated with collagen. Consistent with increased *TGFβ* expression, systemic inflammation resulted in increased LV expression of the collagen gene, *Col1*, and the lysyl oxidase enzyme gene, *LOX*. These results suggest that circulating TNF‐α contributes to local LV inflammation, which increases collagen production and cross‐linking, that may result in fibrosis. Thus, blocking circulating TNF‐α prevents adverse LV remodeling by decreasing local LV inflammation. Systemic inflammation resulted in increased expression of matrix metalloproteinases, suggesting dysregulation of extracellular matrix turnover. Blocking TNF‐α prevented the upregulation of matrix metalloproteinase expression. Despite the reduced collagen formation and collagen turnover in the anti‐TNF‐α group, blocking circulating TNF‐α had no effect on echocardiographic measures of LV relaxation or LV stiffness, suggesting that additional factors that are not regulated by TNF‐α contribute to diastolic dysfunction.

Circulating cytokines such as TNF‐α drive inflammation in a receptor‐mediated manner and exert pleiotropic effects on a number of different cell types (Paulus & Tschope, 2013). These cells, in turn, produce acute phase proteins, such as CRP, vascular adhesion molecules, matrix metalloproteinases, and growth factors (Ma & Xu, 2013; Paulus & Tschope, 2013; Pironti et al., 2018). Etanercept is a fusion protein that inhibits the action of TNF‐α by mimicking the soluble form of the TNF‐α receptor, which binds to TNF‐α in circulation and blunts its effects (Ma & Xu, 2013). In this study, local expression of *IL‐6* in the LV was increased in the CIA group. This suggests that local *IL‐6* expression was upregulated in response to the increased circulating cytokines induced by collagen inoculation. Similarly, a previous study in mice with post‐ischemic heart failure showed increased levels of circulating cytokines that were associated with the upregulation of genes that encode chemokine and cytokine receptors in cardiac tissues (Lachtermacher et al., 2010). The downregulation of cardiac *IL‐6* expression when TNF‐α was blocked suggests that increased circulating TNF‐α increases the local expression of IL‐6. There was no increase in local expression of *TNF‐α* in any of the groups. *TNF‐α* expression is a part of both normal and diseased physiology in the heart (Schumacher & Naga Prasad, 2018). Transcription of *TNF‐α* mRNA is regulated in response to complex signaling systems (Azzawi & Haselton, 1999). Although elevated circulating TNF‐α is considered a biomarker for cardiovascular disease, specific cardiomyocyte expression of *TNF‐α* has been associated with more progressive forms of heart failure including systolic dysfunction, whereby the relative gene dosage is proportional to disease severity (Francis, 1999; Kubota et al., 2001; Lachtermacher et al., 2010). The unchanged *TNF*‐*α* mRNA expression in this model may be explained by the effects of the intervention on the severity of cardiac dysfunction. Indeed, we and others have previously reported early stage diastolic dysfunction or mild cardiovascular disease without systolic impairments in similar models (Le Roux et al., 2021; Mokotedi et al., 2020; Sanghera et al., 2019).

In this study, the increased relative mRNA expression of *CD68* in the CIA group suggests macrophage infiltration of the heart tissues (Franssen et al., 2016). Although the fibroblast‐stimulating growth factor, *TGFβ*, is broadly anti‐inflammatory, it is primarily produced by macrophages (Netea et al., 2017). Thus, the increased expression of *CD68* is consistent with increased expression of *TGFβ* and *IL‐6* in CIA rats, as macrophages are a source of both TGF‐β and IL‐6 (Hulsmans et al., 2018; Witasp et al., 2013). Although it did not reach significance, blocking circulating TNF‐α tended to lower *CD68* mRNA expression in the anti‐TNF‐α group compared with the CIA group. This suggests that circulating TNF‐α may contribute to macrophage infiltration of the heart. Supporting this, blocking soluble TNF‐α has been reported to suppress activation of macrophages in retinal inflammation (Robertson et al., 2003). Consistent with increased relative mRNA expression of *CD68* in the CIA group, we showed increased local expression of *TGFβ* mRNA in this group. Increased expression of pro‐inflammatory cytokines upregulates the expression of *TGFβ* in the heart, which in turn activates cardiac fibroblasts (Villar et al., 2009). Activated cardiac fibroblasts trans‐differentiate into collagen‐producing myofibroblasts, which express α‐smooth muscle actin as they mature (Lacalzada et al., 2011). Despite the upregulation of *TGFβ* mRNA in the CIA group, we did not show any differences in the mRNA expression of α‐SMA. Α‐SMA is upregulated in response to myocardial injury during trans‐differentiation to myofibroblasts, but expression decreases after 21 days (Fu et al., 2018). Therefore, the unchanged α‐SMA expression in this study may be explained by the relatively modest levels of inflammation and the time lapse between inoculation and termination. Therefore, the rate of transition to myofibroblasts may not have been high enough. Consistent with the role of TGF‐β signaling in collagen production and cross‐linking, we showed increased expression of *Col1* and *LOX* in the CIA group and significant associations of the mRNA expression of *TGFβ* with both *Col1*and *LOX* (Goldsmith et al., 2013; Páramo, 2017). Although, increased deposition of collagen I and collagen III in pro‐fibrotic tissues is associated with a less compliant cardiac extracellular matrix, collagen I is more fibrillar and dense in composition compared to the weaker and less mature collagen III (Husse et al., 2007; Querejeta et al., 2004). We also showed increased expression of *Myh7*, which codes for the β‐myosin heavy chain, a well‐known marker of cardiac hypertrophy (Marian, 2021) that has also been localized to areas of fibrosis in the heart (Pandya et al., 2006). However, we showed that only expression of *Col1* was associated with increased RWT. This suggests that fibrosis, rather than hypertrophy, may have contributed to the increased concentric remodeling of the LV (Le Roux et al., 2021). Despite the increased expression of *TGFβ* in the anti‐TNF‐α group, blocking circulating TNF‐α may have partially offset the inflammation‐induced increase in the mRNA expression of *Col1* and *LOX*. This suggests that TNF‐α regulation of collagen deposition and cross‐linking of extracellular matrix components may be independent of the effects of *TGFβ*.

Besides the structural changes of LV geometry and evidence of fibrosis, circulating inflammatory markers impact LV extracellular matrix remodeling by altering the expression of matrix metalloproteinases (Baum & Duffy, 2011). In this study, the local relative mRNA expression of *MMP2* was increased and the expression of *MMP9* tended to be elevated in the CIA group compared to controls. The associations between the mRNA expression of *MMP2* and *IL‐6*, *TGFβ* and *Col1* suggest that *MMP2* is upregulated in response to tissue inflammation and collagen deposition, rather than to an increase in circulating inflammatory markers. This is consistent with previous studies that have shown that tissue inflammation and increased collagen deposition in the extracellular matrix are accompanied by increased expression of MMP2 and MMP9 (Li et al., 2000; Sivasubramanian et al., 2001). The main function of matrix metalloproteinases is the degradation of extracellular matrix components, particularly collagen, to promote extracellular matrix turnover (Liu et al., 2006). Yet, paradoxically, increased matrix metalloproteinase expression is associated with increased, rather than decreased deposition of fibrotic tissue (Li et al., 2000). This is because products of matrix metalloproteinase activity are known to stimulate collagen‐1 synthesis (Li et al., 2000). Consequently, disordered deposition of collagen‐1 disrupts extracellular matrix structure causing fibrosis and LV stiffness (Li et al., 2000). This is consistent with our demonstration that *MMP2* expression is associated with *Col1* expression. Tissue inhibitors of metalloproteinases (TIMPs) inhibit the activity of matrix metalloproteinases (Li et al., 2000). It has been suggested that an increased ratio of matrix metalloproteinases to TIMPs favors fibrosis and LV stiffness (Sivasubramanian et al., 2001). Thus, the increased expression of matrix metalloproteinases that we have shown is likely to override the effects of TIMPs. Taken together, the increased collagen deposition, increased collagen cross‐linking, and increased matrix metalloproteinase expression may have contributed to impaired LV relaxation and increased LV stiffness in the CIA group in this study.

Inflammation‐induced relative expression of *MMP9* and *MMP2* was inhibited in the anti‐TNF‐α group. This suggests that circulating TNF‐α mediates the upregulation of matrix metalloproteinases. Since TNF‐α also mediates the increased expression of the pro‐fibrotic genes *Col1* and *LOX1*, our results show that TNF‐α is likely the cytokine responsible for inflammation‐induced fibrosis and increased relative wall thickness.

We have shown that inflammation increased LV stiffness and impaired LV relaxation and that this was not reversed by blocking circulating TNF‐α (Le Roux et al., 2021). This shows that TNF‐α does not mediate the development of diastolic dysfunction. Since TNF‐α mediates fibrosis, these results show that diastolic dysfunction mostly does not result from fibrosis. Although LV concentric remodeling is one of the main risk factors for LV diastolic dysfunction, others have also shown that LV diastolic dysfunction develops independently of concentric remodeling (Shah, 2013).

A recent study has shown that myocardial infarction caused alterations to elastin, which is another major component of the extracellular matrix that contributes to compliance (Yu et al., 2017). The contribution of elastin to passive stiffness in systemic inflammation requires further investigation. Other molecular pathways independent of extracellular matrix compliance, including cardiomyocyte titin compliance and calcium handling may contribute to the LV diastolic dysfunction in this study (Lewinter & Granzier, 2014; Lipskaia et al., 2010).

This study has some limitations. The inclusion of male and female rats may have reduced statistical power. Pharmacokinetic properties of the anti‐inflammatory drug, which was designed for humans, may have immunogenic effects in rats (Filler et al., 2012). Because etanercept is used to treat rheumatoid arthritis, rather than heart failure, it is possible that it has poor bio‐distribution into cardiac tissues and may not block locally produced TNF‐α (Mann et al., 2008). Although diastolic function was measured using echocardiography in this study, including measures of LV performance assessed by pressure volume loop analysis may have improved the interpretation of the results. Furthermore, performing echocardiography under anesthesia may have impacted echocardiography measures. Lastly, although the presence of inflammatory cells in the heart was supported by mRNA expression of *CD68* and *IL6*, this was not confirmed by histological analysis.

In conclusion, our results show that systemic inflammation impacts macrophage infiltration and local cardiac expression of pro‐fibrotic genes, which promote fibrosis and extracellular matrix remodeling of the LV. Although treatment with etanercept may partially attenuate collagen deposition, collagen turnover, and collagen cross‐linking, it does not prevent the development of diastolic dysfunction. Further investigations are required to determine additional molecular mechanisms of diastolic dysfunction, independent of TNF‐α, in systemic inflammation.

## CONFLICT OF INTEREST

The authors declare that they have no conflict of interest.

## AUTHOR CONTRIBUTIONS

Ashmeetha Manilall: Investigation, validation, writing—original draft preparation, and visualization. Lebogang Mokotedi: Methodology, writing—review and editing. Sulè Gunter: Methodology, writing—review and editing. Regina Le Roux: Investigation, Serena Fourie: Investigation, Colleen A Flanagan: Methodology, validation, writing—review and editing. Aletta Millen: Conceptualization, validation, formal analysis, project administration, funding acquisition, supervision, writing—review and editing.

## Supporting information



Table S1
