# Supplementary material for: Inflammation‐induced left ventricular fibrosis is partially mediated by tumor necrosis factor‐α
Source: Physiol Rep. 2021 Oct 29;9(21):e15062. doi: 10.14814/phy2.15062 (PMC8554769; doi:10.14814/phy2.15062)
Supplement: Supplementary file 1 — Table S1 [file PHY2-9-e15062-s001.docx]

**Supplemental Table 1. Animal characteristics, inflammatory marker concentrations, left ventricular molecular marker expressions and echocardiography variables in male and female rats.**

|  | **Males** | | | **Females** | | |
| --- | --- | --- | --- | --- | --- | --- |
|  | **Control** | **CIA** | **anti-TNF-α** | **Control** | **CIA** | **anti-TNF-α** |
| n | 15 | 13 | 7 | 12 | 15 | 8 |
| Body mass (g) | 590.7 ± 16.7 | 537.7 ± 16.7 | 511.5 ± 22.7* | 320.7 ± 7.1 | 313.3 ± 6.3 | 298.0 ± 8.9 |
| SBP (mm Hg) | 127 ± 3 | 131 ± 3 | 132 ± 4 | 129 ± 2 | 129 ± 2 | 132 ± 3 |
| DBP (mm Hg) | 87 ± 2 | 87 ± 2 | 84 ± 3 | 88 ± 2 | 89 ± 2 | 86 ± 3 |
| **Circulating inflammatory marker concentrations** | | |  |  |  |  |
| TNF-α (pg/ml) | 93.5 ± 9.8 | 153.5 ± 9.8* | 149.9 ± 12.3* | 89.1 ± 10.5 | 158.0 ± 9.3* | 136.7 ± 13.1* |
| IL-6 (pg/ml) | 15.4 ± 2.4 | 27.7 ± 2.4* | 27.5 ± 3.1* | 16.5 ± 3.4 | 32.1 ± 2.9* | 26.1 ± 4.2* |
| CRP (ng/ml) | 0.12 ± 0.05 | 0.64 ± 0.06* | 0.46 ± 0.07* | 0.06 ± 0.06 | 0.47 ± 0.05* | 0.35 ± 0.08* |
| **Left ventricular molecular marker expressions (mRNA)** | | | | | | |
| *TNF-α* | 1.09 (0.91 - 1.39) | 1.17 (0.84 - 1.34) | 1.26 (1.14 - 1.59) | 0.94 (0.69 - 1.18) | 1.02 (0.88 - 1.12) | 1.06 (0.92 - 1.32) |
| *IL-6* | 0.85 (0.52 - 0.86) | 1.48 (1.09 - 1.89)* | 1.04 (0.85 - 1.89) | 0.85 (0.72 - 0.98) | 1.70 (0.81 - 2.22)* | 1.02 (0.86 - 1.28) |
| *CD68* | 1.01 ± 0.08 | 1.23 ± 0.09 | 0.99 ± 0.11 | 0.83 ± 0.06 | 1.00 ± 0.06 | 0.92 ± 0.06 |
| *TGFβ* | 1.25 ±0.06 | 1.46 ± 0.06 | 1.49 ± 0.09 | 1.32 ± 0.07 | 1.45 ± 0.06 | 1.49 ± 0.08 |
| *COL1* | 0.78 ± 0.08 | 1.14 ± 0.07* | 0.98 ± 0.09 | 0.85 ± 0.07 | 1.21 ± 0.06* | 1.02 ± 0.09 |
| *LOX* | 0.92 ± 0.12 | 1.29 ± 0.08* | 1.16 ± 0.13 | 0.82 ± 0.05 | 1.08 ± 0.04* | 0.85 ± 0.06† |
| *αSMA* | 1.08 ± 0.13 | 1.19 ± 0.12 | 0.94 ± 0.18 | 0.90 ± 0.12 | 1.18 ± 0.12 | 0.99 ± 0.14 |
| *Myh7* | 1.06 ± 0.08 | 1.53 ± 0.07 * | 0.93 ± 0.09 † | 0.88 ±0.07 | 0.96 ± 0.06 | 0.89 ± 0.08 |
| *MMP2* | 0.77 ± 0.06 | 0.93 ± 0.06 | 0.81 ± 0.08 | 0.89 ± 0.06 | 1.03 ± 0.06 | 0.83 ± 0.08 |
| *MMP9* | 1.11 ± 0.09 | 1.24 ± 0.10* | 0.64 ± 0.13† | 0.87 ± 0.08 | 1.08 ± 0.08 | 0.60 ± 0.10† |
| **Echocardiography variables** | |  |  |  |  |  |
| RWT | 0.62 ± 0.03 | 0.75 ± 0.03 * | 0.67 ± .04 | 0.62 ± 0.03 | 0.73 ± 0.03* | 0.62 ± 0.04 |
| e' | 4.85 ± 0.13 | 3.65 ± 0.13* | 3.68 ± 0.17* | 4.24 ± 0.16 | 3.58 ± 0.14* | 3.32 ± 0.19* |
| e'/a' | 1.50 ± 0.09 | 1.22 ± 0.09* | 1.14 ± 0.12* | 1.40 ± 0.07 | 1.14 ± 0.06* | 0.94 ± 0.09* |

Data are expressed as means ± SEM or median (IQR). CIA, collagen-induced arthritis, anti-TNF-α, collagen-inoculated and treated with tumour necrosis factor alpha inhibitor, SBP, systolic blood pressure, DBP, diastolic blood pressure, TNF-α, tumour necrosis factor alpha, IL-6, interleukin 6, CRP, C-reactive protein, RWT, relative wall thickness. * p < 0.05 versus control; ^†^ p < 0.05 versus CIA.
